# Osteoarthritis-associated basic calcium phosphate crystals activate membrane proximal kinases in human innate immune cells

**DOI:** 10.1186/s13075-017-1225-0

**Published:** 2017-02-07

**Authors:** Emma M. Corr, Clare C. Cunningham, Laura Helbert, Geraldine M. McCarthy, Aisling Dunne

**Affiliations:** 1 School of Biochemistry & Immunology and School of Medicine, Trinity Biomedical Sciences Institute, Trinity College Dublin, Dublin, Ireland; 2 Mater Misericordiae University Hospital, Dublin, Ireland

**Keywords:** BCP crystals, Inflammation, Syk, PI3K, S100 proteins

## Abstract

**Background:**

Osteoarthritis (OA) is a chronic debilitating joint disorder of particularly high prevalence in the elderly population. Intra-articular basic calcium phosphate (BCP) crystals are present in the majority of OA joints and are associated with severe degeneration. They are known to activate macrophages, synovial fibroblasts, and articular chondrocytes, resulting in increased cell proliferation and the production of pro-inflammatory cytokines and matrix metalloproteases (MMPs). This suggests a pathogenic role in OA by causing extracellular matrix degradation and subchondral bone remodelling. There are currently no disease-modifying drugs available for crystal-associated OA; hence, the aim of this study was to explore the inflammatory pathways activated by BCP crystals in order to identify potential therapeutic targets to limit crystal-induced inflammation.

**Methods:**

Primary human macrophages and dendritic cells were stimulated with BCP crystals, and activation of spleen tyrosine kinase (Syk), phosphoinositide-3 kinase (PI3K), and mitogen-activated protein kinases (MAPKs) was detected by immunoblotting. Lipopolysaccharide (LPS)-primed macrophages were pre-treated with inhibitors of Syk, PI3K, and MAPKs prior to BCP stimulation, and cytokine production was quantified by enzyme-linked immunosorbent assay (ELISA). Aa an alternative, cells were treated with synovial fluid derived from osteoarthritic knees in the presence or absence of BCP crystals, and gene induction was assessed by real-time polymerase chain reaction (PCR).

**Results:**

We demonstrate that exposure of primary human macrophages and dendritic cells to BCP crystals leads to activation of the membrane-proximal tyrosine kinases Syk and PI3K. Furthermore, we show that production of the pro-inflammatory cytokines interleukin (IL)-1α and IL-1β and phosphorylation of downstream MEK and ERK MAPKs is suppressed following treatment with inhibitors of Syk or PI3K. Finally, we demonstrate that treatment of macrophages with BCP crystals induces the production of the damage-associated molecule S100A8 and MMP1 in a Syk-dependent manner and that synovial fluid from OA patients together with BCP crystals exacerbates these effects.

**Conclusions:**

We identify Syk and PI3K as key signalling molecules activated by BCP crystals prior to inflammatory cytokine and DAMP expression and therefore propose that Syk and PI3K represent potential targets for the treatment of BCP-related pathologies.

## Background

It is well established that intra-articular deposition of particulates, such as gout-associated monosodium urate (MSU) crystals and osteoarthritis (OA)-associated basic calcium phosphate (BCP) crystals, drives joint degeneration through the production of pro-inflammatory cytokines and cartilage-degrading proteases. BCP crystals are a heterogeneous group of ultramicroscopic crystalline substances composed mainly of hydroxyapatite (HA), along with smaller proportions of its precursor forms octacalcium phosphate (OCP) and tricalcium phosphate [1, 2]. The concentration of BCP crystals found in the synovial fluid is reported to be between 20 and 100 μg/ml [3–5]. Furthermore, a 3-year prospective analysis of synovial fluid (SF) samples obtained from 330 patients with knee OA showed that the initial presence of BCP crystals was associated with worsening of radiographic lesions [6]. The concentration in the tissues is more difficult to quantify; however, Fuerst and colleagues demonstrated that human knee and hip cartilage specimens (*n* = 120 and *n* = 80, respectively), harvested at the time of total joint arthroplasty for primary OA, contained BCP crystals in 100% of cases [7, 8]. Sun et al. have also reported calcium deposition in all eight OA menisci harvested at the time of joint replacement surgery and that calcium crystal formation could be generated by both the meniscal and cartilage cells of patients with end-stage OA [9]. As crystal deposition does not occur in healthy cartilage, it is becoming more widely accepted that cartilage calcification plays a pathogenic role in OA and that BCP crystals are not the “innocent bystanders” that they were once believed to be. Indeed, the crystals are now considered to be a damage-associated molecular pattern (DAMP) as they can activate fibroblasts through a variety of signalling pathways involving protein kinase C (PKC), ERK1/2 mitogen-activated protein kinases (MAPKs), and transcription factors such as NFκB which, in turn, leads to the production of tumour necrosis factor (TNF)α, interleukin (IL)-6, and IL-1β [10, 11]. Cytokine induction has also been observed in BCP-activated chondrocytes [12] and macrophages [13–16], with IL-1β, in particular, implicated as a key player in the inflammatory and degradative responses observed in OA joints. It is induced early on in the disease and has the ability to upregulate matrix metalloproteases (MMPs) and aggrecanases, induce cell infiltration into the joints, promote osteoclastogenesis, and suppress the biosynthesis of type II collagen and aggrecan, crucial components of cartilage [17–20]. We have recently demonstrated that BCP crystals can inhibit anti-osteoclastogenic cytokine signalling, and therefore the crystals may also contribute to joint destruction by promoting the differentiation of bone-resorbing osteoclasts [21]. Together, these events lead to an imbalance in the production of anabolic versus catabolic mediators and, in many cases, total joint replacement is eventually required.

Current therapies for OA focus merely on pain relief and improvement of joint function rather than halting disease progression. While much progress has been made in elucidating the cellular and molecular events contributing to OA, the complex nature of the disease has hampered the development of a successful disease-modifying drug despite a multitude of potential targets. We and others have previously reported that BCP crystals activate the NLRP3 inflammasome in vitro leading to potent IL-1β production. However, conflicting results from in vivo models have called into question the relevance of these findings to a clinical setting, and additional targets are currently being sought [14, 16, 22]. Another potential target of interest in particulate-mediated disease is the membrane-proximal kinase, spleen tyrosine kinase (Syk). Belonging to the tyrosine kinase family, Syk is activated during phagocytosis and following Fc receptor engagement on immune cells [23]. Syk has been implicated in both the internalisation of MSU crystals and subsequent MSU-induced signalling in neutrophils and dendritic cells (DC) [24, 25]. In addition to MSU crystals, alum particles and cholesterol crystals have also been reported to activate Syk in a receptor-independent manner, by a process known as membrane affinity-triggered signalling (MATS). This involves direct binding of the particulates to the cell membrane which results in lipid raft formation and aggregation of immunoreceptor tyrosine-based activation motif (ITAM)-containing molecules which mediate the recruitment of Syk to the plasma membrane and its subsequent activation [26, 27]. We have previously demonstrated that BCP crystals activate Syk and its downstream interacting partner, phosphoinositide-3 kinase (PI3K), in murine macrophages [16]. Therefore, the aim of this study was to determine whether BCP crystals activate similar pathways in human macrophages and DC and to examine the downstream effect of inhibiting these pathways in order to identify potential therapeutic targets to treat crystal-induced inflammation in OA.

## Methods

### Reagents

Ultrapure lipopolysaccharide (LPS) and the PI3K inhibitor, LY294002, were from Invivogen (Toulouse, France). The Syk inhibitor, R788, was from AdooQ BioScience (Irvine, CA, USA). Recombinant human M-CSF was from PeproTech (Rocky Hill, NJ, USA). Recombinant human IL-4 and GM-CSF were from Immunotools (Friesoythe, Germany). Lymphoprep was from Stemcell Technologies (Grenoble, France). Primary antibodies were obtained from Cell Signaling Technology (Beverly, MA, USA). The Syk inhibitor, piceatannol, methyl-β-cyclodextrin (M-βCD), secondary antibodies, cell culture reagents and all other chemicals were from Sigma Aldrich (St. Louis, MO, USA). Human FcR binding inhibitor was from eBioscience. BCP crystals were synthesized by alkaline hydrolysis of brushite as described previously [28] and contain partially carbonate-substituted hydroxyapatite in addition to octacalcium phosphate. OA synovial fluid was obtained with permission from The Mater Misericordiae Hospital Research Ethics Committee.

### Cell culture and differentiation

Peripheral blood mononuclear cells (PBMCs) were isolated by means of density gradient centrifugation from leukocyte-enriched buffy coats from anonymous healthy donors, obtained with permission from the Irish Blood Transfusion Board, St. James’s Hospital, Dublin. CD14^+^ cells were positively selected using anti-CD14 magnetic beads (Miltenyi Biotech, Germany) and shown to be >90% pure, as determined by flow cytometry. Cells were cultured for 6 days in six-well plates (for immunoblotting assays) or 24-well plates (for real-time polymerase chain reaction (PCR) and enzyme-linked immunosorbent assay (ELISA)) in RPMI 1640 medium supplemented with 1% penicillin-streptomycin and 10% foetal bovine serum. Cells were treated on days 0 and 3 with M-CSF (50 ng/ml) for macrophage differentiation or IL-4 (40 ng/ml) and GM-CSF (50 ng/ml) for DC differentiation (as adapted from [29, 30]). Cells were shown to be >95% pure as determined by flow cytometry, using CD14 and CD11b as macrophage markers and CD14 and CD209 as DC markers [31, 32].

#### Kinase activation

Primary macrophages or DC (2 × 10^6^/well) were stimulated with BCP crystals (50 μg/ml) over the course of 30 min. Alternatively, cells were pre-treated with piceatannol, R788, or LY294002 for 30 min prior to crystal stimulation. Cells were lysed by the addition of RIPA buffer (Tris 50 mM; NaCl 150 mM; SDS 0.1%; sodium deoxycholate 0.5%; Triton X 100) containing phosphatase inhibitor cocktail 3 (Sigma-Aldrich). Samples were electrophoresed on a 12% SDS-polyacrylamide gel and transferred to PVDF membranes prior to detection with anti-phospho-Syk, anti-phospho-PI3K, anti-phospho-MEK, or anti-phospho-ERK.

#### Cytokine measurements

Primary macrophages (5 × 10^5^/well) were primed with a known TLR4 activator, LPS (100 ng/ml), for 2 h prior to treatment with piceatannol, LY294002, M-βCD, or a human FcR binding inhibitor for 1 h and stimulation with BCP crystals (50 μg/ml) for 6 h. Supernatants were harvested and cytokine concentrations were quantified by ELISA (R&D Systems). S100A8 is also capable of activating TLR4; therefore, in order to determine whether this DAMP could be used to facilitate pro-IL-1β induction, in place of LPS, primary macrophages (5 × 10^5^/well) were primed with endotoxin-free recombinant S100A8 (1 μg/ml) for 3 h prior to stimulation with BCP crystals (50 μg/ml) for 6 h, and cytokine concentrations were quantified by ELISA.

#### Real-time PCR

Human macrophages (5 × 10^5^/well) were treated with BCP crystals alone for 3, 6, or 24 h or were pre-treated with piceatannol (50 μM, 100 μM) or LY294002 (25 μM, 50 μM) prior to BCP crystal treatment for 6 h. Alternatively, human macrophages were treated with OA synovial fluid alone or in combination with BCP crystals for 24 h. RNA was extracted using High Pure RNA Isolation Kits (Roche) and assessed for concentration and purity using the NanoDrop 2000c UV-Vis spectrophotometer. RNA was equalised and reverse transcribed using the Applied Biosystems High-Capacity cDNA reverse transcription kit. Real-time PCR was carried out on triplicate cDNA samples with the use of the CFX96 Touch Real-Time PCR Detection System (Bio-Rad Laboratories, CA, USA). Reactions included TaqMan fast universal PCR Master Mix (Applied Biosystems), cDNA and predesigned TaqMan S100A8, and MMP1 and TIMP1 gene expression probes (Applied Biosystems). mRNA expression data were normalised to the housekeeping gene, 18S ribosomal RNA, and relative gene expression levels were analysed using the 2^−ΔΔCT^ method.

### Statistical analysis

All experiments were run at least three times. For real-time PCR and ELISA, three technical replicates, per donor, were obtained and the mean for each donor ± SEM was then plotted for *n* ≥ 3 healthy donors. Statistical analysis was performed by one-way analysis of variance (ANOVA) with Tukey post-test where applicable or Student’s *t* test when comparing only two observations. All experiments were run at least three times and analysed on GraphPad Prism 6 software. A *P* value ≤ 0.05 was deemed statistically significant.

## Results

### BCP crystals activate Syk and PI3K in primary human macrophages and DC

**Fig. 1:**
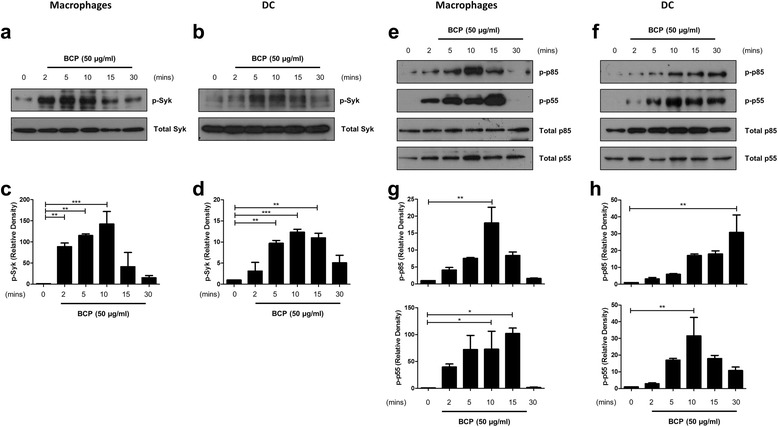
BCP crystals activate Syk and PI3K in primary macrophages and DC. **a**, **e** Human macrophages and **b**, **f** DC (2 × 10^6^ cells/well) were stimulated with BCP crystals (50 μg/ml) for the indicated time points, and phosphorylation of Syk and PI3K was detected by immunoblotting using phospho-specific antibodies. Representative blots of three independent experiments are shown. **c**, **d**, **g**, **h** Densitometric analysis of three blots was performed using ImageJ software. Bar graphs illustrate the mean (± SEM) increase in phosphorylation, relative to the untreated sample (0) and normalised to total Syk/PI3K protein. *P ≤ 0.05, **P ≤ 0.01, ***P ≤ 0.001

We have previously demonstrated that BCP crystals activate the membrane-proximal tyrosine kinase Syk in murine bone marrow-derived macrophages (BMDM) [16]. It is also reported to be activated by MSU crystals, alum particles, and cholesterol crystals [24–27]. In order to determine if BCP crystals can induce the activation of Syk in human innate immune cells, primary macrophages and DC were stimulated with BCP crystals (50 μg/ml) over the course of 30 min. This concentration of BCP crystals was chosen as it is within the range used in previously published studies [14, 15, 22] and is considered to be physiologically relevant as the concentration in OA synovial fluid ranges between 20 and 100 μg/ml [3–5]. Activation of Syk, as indicated by phosphorylation, was examined by immunoblotting. Phosphorylation of Syk was detected in both macrophages (Fig. 1a) and DC (Fig. 1b) within 2 min of BCP crystal treatment and increased in the first 10 min. Densitometric analysis of three Western blots revealed that maximal phosphorylation occurs at 10 min post-stimulation in both cell types (Fig. 1c and d).

Having demonstrated that BCP crystals are capable of activating Syk in both primary human macrophages and DC, we next sought to determine whether PI3K (a known interacting partner) is activated by BCP crystals downstream of Syk. Robust phosphorylation of the regulatory p85 and p55 subunits of the enzyme was detected in both macrophages (Fig. 1e) and DC (Fig. 1f) within 10 min of crystal treatment, with densitometric analysis of three Western blots revealing that maximal phosphorylation occurs at between 10 and 30 min in both cell types (Fig. 1g and h).

### BCP crystals drive IL-1 production by primary macrophages in a Syk- and PI3K-dependent manner

**Fig. 2:**
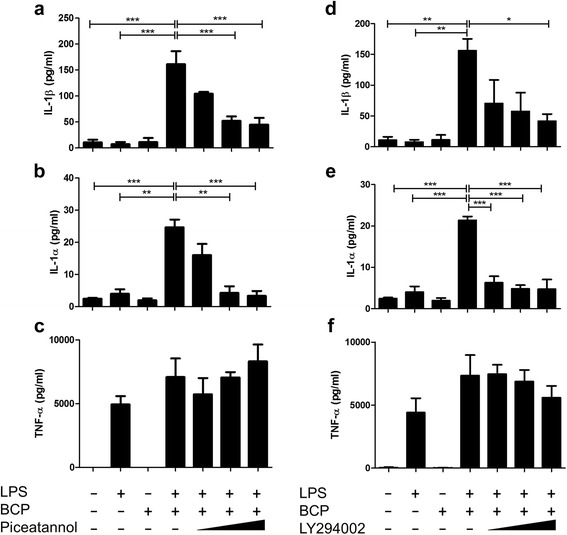
Inhibition of Syk and PI3K reduces BCP crystal-induced IL-1 production in primary macrophages. Human macrophages (0.5 × 10^6^ cells/well) were primed with LPS (100 ng/ml) for 2 h prior to treatment with **a**–**c** piceatannol (10 μM, 25 μM, and 50 μM) or **d**–**f** LY294002 (10 μM, 25 μM, and 50 μM) for 1 h and stimulation with BCP crystals (50 μg/ml) for 6 h. Cell supernatants were assessed for IL-1β, IL-1α, and TNF-α by ELISA. Results indicate mean (± SEM) of three independent experiments. *P ≤ 0.05, **P ≤ 0.01, ***P ≤ 0.001 vs LPS + BCP

BCP crystals are known to drive IL-1β production in murine macrophages that have been primed, for example, with a TLR agonist such as LPS [14–16]. Priming serves to induce pro-IL-1β expression within the cell while subsequent treatment of cells with the crystals leads to IL-1β processing and secretion. In an in vitro setting, this event is mediated by the NLRP3 inflammasome and active caspase-1, whereas it is likely to be mediated by alternative enzymes such as granzymes in the diseased joint [15]. In order to investigate if Syk is involved in BCP crystal-induced IL-1 production, primary human macrophages were primed with LPS (100 ng/ml) for 2 h prior to treatment with piceatannol, a pharmacological inhibitor of Syk, for 1 h and stimulation with BCP crystals for 6 h. Treatment with the Syk inhibitor resulted in a dose-dependent reduction in BCP crystal-induced IL-1β production, while having no effect on LPS-induced TNF-α which occurs independently of caspase-1 activation (Fig. 2a and c). Syk inhibition also significantly reduced IL-1α secretion (Fig. 2b) which is known to coincide with IL-1β release during pyroptosis. Similar results were obtained with an additional Syk inhibitor, R788, which is orally available (data not shown).

In order to determine the effect of PI3K inhibition on BCP crystal-induced IL-1 production, LPS-primed human macrophages were pre-treated with the PI3K inhibitor, LY294002, for 1 h prior to BCP crystal stimulation. Similar to Syk inhibition, PI3K inhibition significantly reduced both IL-1α and IL-1β secretion, once again having no effect on LPS-induced TNFα production (Fig. 2d–f). This suggests that activation of Syk and PI3K is directly coupled to BCP crystal-induced cytokine production.

### BCP crystal-induced IL-1 production occurs via lipid raft formation in primary human macrophages

**Fig. 3:**
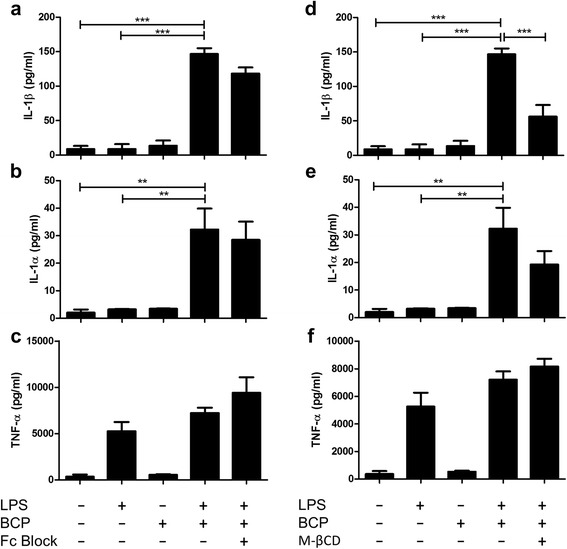
BCP crystal-induced IL-1 production occurs via lipid raft formation in primary macrophages. LPS-primed human macrophages were treated with **a**–**c** an Fc receptor blocking antibody (5 μg/ml) or **d**–**f** M-βCD (10 mM) for 1 h prior to BCP crystal stimulation for 6 h. Cell supernatants were assessed for IL-1β, IL-1α, and TNF-α by ELISA. Results indicate mean (± SEM) of three independent experiments. ***P < 0.001

Syk is typically activated following FcγR engagement which induces ITAM phosphorylation [23]; however, recent studies have demonstrated that MSU crystals, cholesterol crystals, and alum particles are capable of directly binding to the plasma membrane and activating Syk in a receptor-independent manner via MATS [24–27]. In order to ascertain whether MATS or FcγR activation is involved in BCP crystal-induced IL-1 production, LPS-primed human macrophages were either treated with an FcγR neutralising antibody or depleted of membrane cholesterol with M-βCD (10 mM) to prevent lipid sorting, prior to stimulation with BCP crystals. Blockade of the FcγR had no effect on BCP crystal-induced IL-1 production (Fig. 3a and b) whereas treatment with M-βCD significantly reduced BCP crystal-induced IL-1β production (Fig. 3d) and, while not statistically significant, also reduced IL-1α (Fig. 3e). Neither FcγR blockade nor M-βCD treatment affected LPS-induced TNFα (Fig. 3c and f). These results suggest that, like alum particles, MSU, and cholesterol crystals, BCP crystals may activate cell signalling pathways and drive cytokine production by primary macrophages through direct interaction with the plasma membrane.

### BCP crystals activate MEK and ERK MAPK in primary human macrophages and DC

**Fig. 4:**
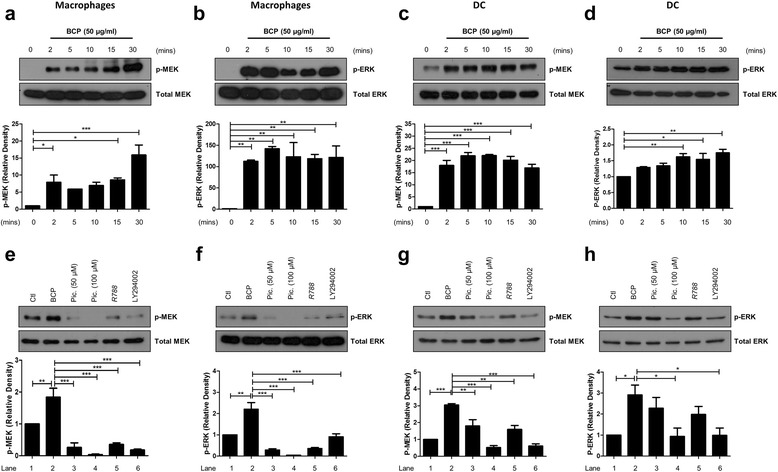
BCP crystals activate MEK and ERK downstream of Syk/PI3K in primary macrophages and DC. **a**,**b** Human macrophages and **c**, **d** DC (2 × 10^6^ cells/well) were stimulated with BCP crystals (50 μg/ml) for the indicated time points. Phosphorylation of MEK and ERK was detected by immunoblotting using phospho-specific antibodies. **e**,**f** Human macrophages and **g**,**h** DC were pre-treated with piceatannol (50 μM, 100 μM; lanes 3, 4), R788 (5 μM; lane 5), or LY294002 (50 μM; lane 6) for 30 min prior to stimulation with BCP crystals for 15 min. Phosphorylation of MEK and ERK was detected by immunoblotting. Representative blots of three independent experiments are shown. Densitometric analysis of three blots was performed using ImageJ software. Bar graphs illustrate the mean (± SEM) increase in phosphorylation, relative to the untreated sample (0) and normalised to total MEK/ERK protein. *P ≤ 0.05, **P ≤ 0.01, ***P ≤ 0.001

It well known that MEK and ERK MAPK are activated downstream of Syk and PI3K [33]. In addition, BCP crystals have been shown to activate MAPKs in human fibroblasts and osteoclast precursor cells [16, 21, 34]. In order to determine whether BCP crystals can activate MAPKs in human innate immune cells, and to determine if this is associated with the activation of membrane-proximal kinases, primary macrophages and DC were stimulated with BCP crystals over the course of 30 min and MEK/ERK activation, as indicated by phosphorylation, was assessed by immunoblotting. Robust BCP crystal-induced phosphorylation of MEK and ERK was evident after 2 min in macrophages (Fig. 4a and b; corresponding densitometry is also shown). While basal phosphorylation of MEK and ERK was higher in DC than in macrophages, densitometric analysis of three Western blots from individual donors revealed that phosphorylation of both kinases was significantly increased following BCP treatment (Fig. 4c and d; corresponding densitometry is also shown).

In order to confirm that BCP crystal-induced MEK/ERK activation occurs downstream of Syk and PI3K, primary human macrophages and DC were pre-treated for 30 min with one of two Syk inhibitors, piceatannol (50 μM or 100 μM) or R788 (5 μM), or the PI3K inhibitor, LY294002 (50 μM), prior to stimulation with BCP crystals for 15 min. As previously observed, BCP crystals induced the phosphorylation of MEK and ERK in primary macrophages (Fig. 4e and f; corresponding densitometry is also shown) and DC (Fig. 4g and h; corresponding densitometry is also shown) and this was reduced when either Syk or PI3K were inhibited, which demonstrates that BCP crystals activate MAPKs in a Syk- and PI3K-dependent manner.

### BCP crystals upregulate the damage-associated molecules, S100A8, S100A12, and MMP1 in primary macrophages in a Syk- and PI3K-dependent manner

**Fig. 5:**
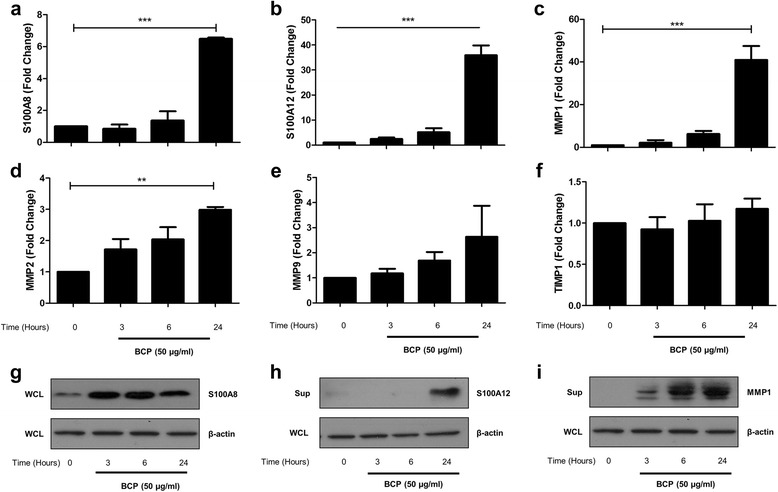
BCP crystals induce expression of damage-associated molecules and MMPs in primary human macrophages in a Syk- and PI3K-dependent manner. Human macrophages (0.5 × 10^6^ cells/well) were stimulated with BCP crystals (50 μg/ml) for 3, 6, and 24 h, and mRNA levels of **a** S100A8, **b** S100A12, **c** MMP1, **d** MMP2, **e** MMP9, and **f** TIMP1 were analysed by real-time PCR. Alternatively, supernatants were harvested; whole cell lysates (WCL) were prepared and both were analysed for the presence of **g** S100A8, **h** S100A12, and **i** MMP1 protein by immunoblotting. Representative blots of three independent experiments are shown. *P ≤ 0.05, **P ≤ 0.01, ***P ≤ 0.001

The calgranulins, specifically S100A8 and S100A9, have been implicated in both rheumatoid arthritis and OA and are considered potent damage-associated molecules which exacerbate inflammation following their release under conditions of cellular stress or injury [35, 36]. In a collagenase-induced OA model, S100A8 and S100A9 expression remained elevated long after inflammatory cytokine levels had subsided, while in a BCP crystal-induced peritonitis model, both peritoneal and serum concentrations of S100A8 and S100A9 were increased [35–37]. Studies from our laboratory have demonstrated that BCP crystals can directly induce the upregulation of S100A8 in murine macrophages [16]; therefore, experiments were next carried out in order to determine whether these crystals also induce the expression of this DAMP in human immune cells. We also examined expression of the DAMP, S100A12, which, although not as widely studied as S100A8, has also been implicated in OA [38]. Primary human macrophages were stimulated with BCP crystals for 3, 6, and 24 h, and expression of S100A8 and S100A12 was analysed by real-time PCR. A time-dependent increase in S100A8 and S100A12 mRNA was observed in response to BCP crystal treatment, with significant induction observed at 24 h post-stimulation (Fig. 5a and b). We also examined the expression of MMP1, MMP2, and MMP9 given their known association with cartilage destruction in OA [39, 40]. As with the S100 proteins, MMP1 mRNA was significantly elevated after 24 h of crystal treatment (Fig. 5c). There was also a modest increase in MMP2 (Fig. 5d), whereas there was no significant effect on MMP9 or the MMP inhibitor, TIMP1 (Fig. 5e and f). An increase in S100A8 protein expression was also detected in cell lysates after 3 h, suggesting a rapid mRNA turnover (Fig. 5g), while a time-dependent increase in the secreted and active forms of MMP1 and S100A12 was detected in the cell supernatants (Fig. 5h and i), which may further exacerbate damage at a site of inflammation.

**Fig. 6:**
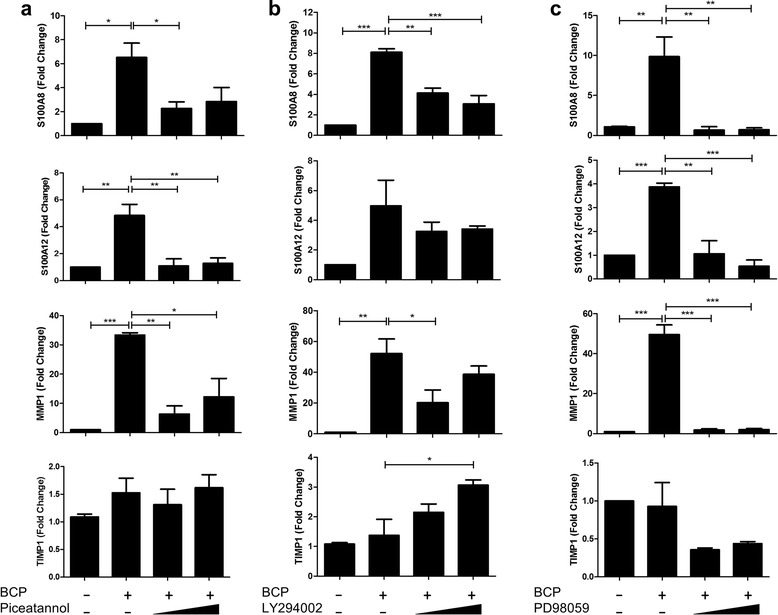
BCP crystals induce expression of S100A8, S100A12, and MMP1 in primary macrophages in a Syk-, PI3K-, and ERK-dependent manner. Human macrophages were pre-treated with **a** piceatannol (50 μM, 100 μM), **b** LY294002 (25 μM, 50 μM), or **c** PD98059 (5 μM, 10 μM) for 30 mins prior to stimulation with BCP crystals for 24 h. mRNA levels of S100A8, S100A12, MMP1, and TIMP1 were analysed by real-time PCR. Results indicate mean (± SEM) of three independent experiments. *P ≤ 0.05, **P ≤ 0.01, ***P ≤ 0.001

We next examined the effect of Syk, PI3K, and MAPK inhibition on the induction of S100A8, S100A12, and MMP1 by BCP crystals. Macrophages were pre-treated with the Syk inhibitor, piceatannol, the PI3K inhibitor, LY294002, or the ERK/MEK inhibitor, PD98059, for 30 min prior to BCP crystal treatment for 24 h, and expression of S100A8, S100A12, MMP1, and TIMP1 was analysed by real-time PCR. All three inhibitors significantly reduced BCP crystal-induced S100A8, S100A12, and MMP1 expression (Fig. 6a–c) suggesting that all three signalling molecules are involved in their induction. The ERK inhibitor reduced basal TIMP1 expression; however, this was not significant, while the PI3K inhibitor appeared to enhance the expression of TIMP1 which reflects previous reports that inhibition of PI3K correlated with induction of TIMP1 [41, 42].

**Fig. 7:**
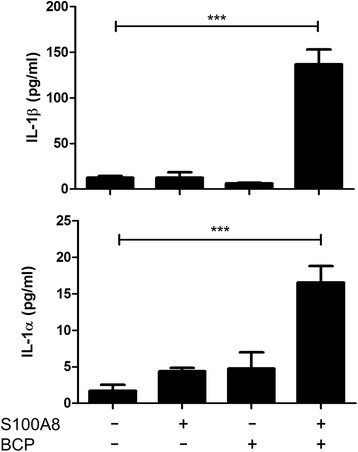
S100A8 primes human macrophages for BCP-induced IL-1β production. Human macrophages were primed with recombinant human S100A8 (1 μg/ml) for 3 h prior to stimulation with BCP for 6 h. Cell supernatants were assessed for IL-1β, IL-1α, and TNF-α by ELISA. Results indicate mean (± SEM) of three independent experiments. ***P ≤ 0.001

As mentioned previously, an initial priming signal is required to drive pro-IL-1β expression prior to its subsequent proteolytic processing and release. The standard in vitro system utilises LPS for priming cells; however, this is unlikely to act as signal 1 in the context of OA and it has been suggested that DAMPs released at the site of injury can assume this role in vivo. Given that S100A8 is capable of activating TLR4 and is shown here to be induced by BCP crystals, we next examined if substitution of endotoxin-free recombinant S100A8 for LPS was sufficient to facilitate pro-IL-1β induction prior to BCP-induced processing in vitro. Primary human macrophages were primed with S100A8 for 3 h and then stimulated with BCP crystals for 6 h, and cytokine production was quantified. Both IL-1β and IL-1α were found to be significantly elevated following S100A8 priming and BCP stimulation (Fig. 7), suggesting that the DAMP can indeed induce pro-IL-1β expression. As expected, given that S100A8 is a TLR4 ligand, TNFα was also induced following S100A8 treatment (data not shown). Taken together, these results suggest that, in addition to pro-inflammatory cytokine production, Syk and PI3K are involved in BCP crystal-induced S100A8, S100A12, and MMP1 induction, making them attractive therapeutic targets.

### OA synovial fluid enhances BCP crystal-induced S100A8 and MMP1 production in a Syk-dependent manner

**Fig. 8:**
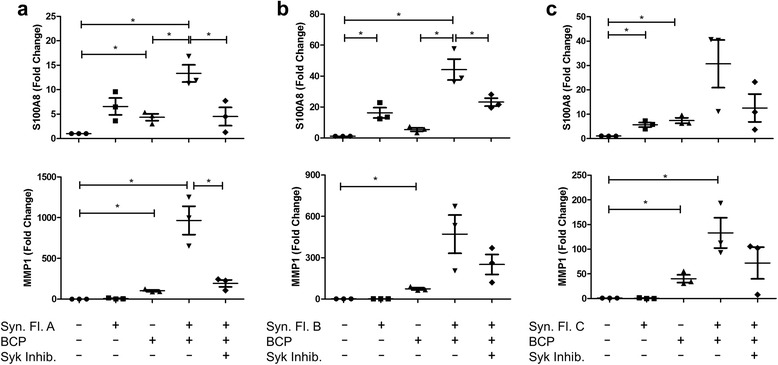
BCP crystals exert synergistic effects with OA synovial fluid on primary macrophages. Human macrophages (0.5 × 10^6^ cells/well) were pre-treated with R788 (2.5 μM) prior to treatment with BCP crystals alone, synovial fluid from one of three OA patients (**a**, **b** or **c**), or OA synovial fluid and BCP crystals together for 24 h. Expression of (*upper panel*) S100A8 and (*lower panel*) MMP1 were analysed by real-time PCR. Results indicate mean (± SEM) of at least three healthy macrophage donors treated with synovial fluid from one OA patient. *P ≤ 0.05, **P ≤ 0.01

Having observed that OA-associated BCP crystals drive S100A8, S100A12, and MMP1 expression in human innate immune cells, we next sought to examine 1) whether synovial fluid from OA patients exerts a similar effect to the crystals, and 2) whether BCP crystals and OA synovial fluid synergise to promote cartilage damage. Primary human macrophages from healthy donors were treated with synovial fluid from one of three OA patients (A, B, or C) either alone or in the presence of BCP crystals for 24 h, and expression of S100A8 and MMP1 was analysed by real-time PCR (Fig. 8a–c). As previously observed, BCP crystals alone upregulated the expression of both S100A8 and MMP1. Using a Student’s paired *t* test to analyse the effect of OA synovial fluid alone versus no treatment, expression of S100A8 was significantly increased as previously observed; *P* values: A = 0.04, B = 0.06, C = 0.02. Interestingly, co-treatment with OA synovial fluid and BCP crystals led to higher levels of both S100A8 and MMP1 when compared with either synovial fluid or BCP crystals alone. Furthermore, pre-treatment of macrophages with the Syk inhibitor R788 dampened the synergistic effects observed following co-treatment, suggesting that R788 is effective in the presence of synovial fluid, which is important when considering the optimum method of administration of an OA drug.

## Discussion

BCP crystals are associated with a number of rheumatic syndromes, and particularly with OA where the HA form of these crystals is most prevalent and is found to be deposited in the joints of 70% of total OA cases and in 100% of knee and hip osteoarthritic joints requiring arthroplasty. The crystals can be found in the synovial fluid and cartilage and are thought to form as a result of dysregulated ossification processes. They are known to play a pathogenic role in OA through the activation of macrophages, synovial fibroblasts, and articular chondrocytes [3, 7, 22, 43–45]. This study provides further insight into the mechanism by which BCP crystals activate intracellular signalling pathways in human innate immune cells and induce pro-inflammatory cytokine production. We report that BCP crystals activate the membrane-proximal kinase Syk and its interacting partner PI3K, and demonstrate that pharmacological inhibition of these enzymes abrogates BCP-induced IL-1α and IL-1β production. We demonstrate that, like gout-associated MSU crystals, alum particles, and cholesterol crystals, BCP crystals also activate Syk by MATS. In all cases, disruption of lipid rafts with M-βCD prevents the ITAM phosphorylation which normally ensues following the direct binding of the particulates to the cell membrane and thus prevents the recruitment and activation of Syk [24–27]. Furthermore, this results in a significant impairment of IL-1 production. Atomic Force Microscopy could be used for a more in-depth analysis to confirm that BCP crystals are, indeed, mediating their effects on macrophages through a direct interaction with the cell membrane. In addition to driving cell activation, this interaction is likely to cause cell damage, leading to the release of DAMPs which may propagate the initial inflammatory response. Interestingly, phosphorylation of the two subunits of PI3K appears to be regulated differently by BCP crystals, and further study is required to determine if these events are mutually exclusive. We have also shown that BCP crystals activate MEK and ERK MAPK downstream of Syk and PI3K, thus providing a link between the initial immune cell activation and subsequent gene expression. The PI3K pathway is a potential target for OA treatment as it can regulate gene expression through its target proteins which include NF-κB, a transcription factor that is reported to regulate MMP production [46, 47]. We demonstrate that BCP crystals directly induce the upregulation of the calgranulins S100A8 and S100A12 as well as the cartilage-degrading protease MMP1 in a Syk- and PI3K-dependent manner, and report that the presence of OA synovial fluid exacerbates the effects of BCP crystals on macrophages.

S100A8 was first identified in the context of RA where both the serum and synovial concentration of it and its binding partner, S100A9, were found to be elevated in patient samples [48]. Recent studies using the collagenase-induced OA model subsequently revealed a potential catabolic role for these proteins in OA-associated inflammation, revealing that S100A8 was abundant in the synovium of both early-stage and late-stage OA patients [35]. Of particular interest was the observation that serum levels of S100A8 were markedly higher in patients showing progression of OA, rather than those without progression, suggesting that these proteins may also be useful markers of disease advancement. Interestingly, analysis of cartilage from OA patients revealed that the protein is found only in areas of proteoglycan loss, suggesting a correlation between the presence of S100A8/A9 and MMP activity leading to aggrecan degradation [49]. The same study demonstrated that stimulation of cartilage explants with S100A8 and S100A9, or a combination of the two, led to increased mRNA levels of MMPs 1, 9, and 13, as well as IL-6, IL-8, and monocyte chemotactic protein-1, while it significantly downregulated the expression of aggrecan and type II collagen. A role for S100A12 has also been implicated in OA, where immunohistochemical analyses revealed that S100A12 expression was markedly increased in OA cartilage compared to non-OA cartilage. The same study also demonstrated that mRNA expression of S100A12 was significantly upregulated in OA chondrocytes in vitro by IL-1β stimulation and that treatment of OA chondrocytes with recombinant human S100A12 resulted in a significant increase in MMP13 mRNA expression [38]. This suggests that S100 proteins may amplify the inflammatory response and induce the degradation of cartilage, while also preventing its repair and regeneration. TLR4 has been identified as the key receptor for S100A8/9 activity, and we have demonstrated that S100A8 can act as a priming signal for BCP crystal-induced IL-1β maturation (at least in an in vitro context). Hence, small molecule inhibitors or biologics targeting TLR4 and/or relevant S100 proteins may be of benefit to OA patients. Furthermore, Syk and PI3K appear to be activated upstream of gene transcription as pharmacological inhibition of these molecules reduces BCP crystal-induced S100A8 and MMP1 mRNA. Therefore, Syk and PI3K may also represent potential therapeutic targets. Indeed, preventing the actual deposition of calcium crystals, as recently demonstrated in a murine OA model by Nasi et al. [50], could limit these responses in the first instance and prevent crystal-associated cell inflammation. Expression of Syk and PI3K has been detected in the intimal lining of OA synovial tissues at similar levels to those detected in healthy synovial tissue, though the degree of activation compared to healthy synovial tissue has not been investigated in any detail [51, 52]. Furthermore, Syk is also known to be expressed by osteoclasts and is crucial to osteoclastic bone resorption [53]; therefore, further studies are required to determine if Syk activation is heightened in OA joints.

## Conclusions

The studies presented here demonstrate that BCP crystals are capable of activating specific intracellular signalling pathways which drive inflammation and the production of cartilage-degrading enzymes and DAMPs that promote disease initiation and exacerbate progression in OA. Patients are currently treated with intra-articular corticosteroids and non-steroidal anti-inflammatory drugs to provide symptomatic relief. Corticosteroids have previously been demonstrated to reduce early OA changes such as osteophyte formation and cartilage lesion in a canine OA model [54] and to reduce cartilage degradation when administered early after anterior cruciate ligament injury in a porcine model [55]; however, they do not entirely halt disease progression. Clinical trials are currently underway to examine the effect of intra-articular administration of a synthetic glucocorticoid on pain, joint function, inflammation, and cartilage degradation (ClinicalTrials.gov identifier: NCT01692756). Anti-cytokine, anti-MMP therapies, and anti-S100A8/A9 therapies are promising candidates for new disease-modifying OA drugs. However, preventing the expression and/or release of degradative mediators rather than inhibiting their activity may prove more efficient. Membrane-proximal kinases such as Syk and PI3K require consideration not just in the context of OA but also for other crystal-mediated diseases such as gout and atherosclerosis, and in vivo studies are required in order to fully implicate these kinases in crystal-induced responses. The orally available Syk inhibitor R788 that was used in this study has previously shown efficacy in clinical trials for rheumatoid arthritis; however, reports of side effects during phase III trials led to trial termination [56]. Nevertheless, a modification on the current drug or, indeed, an alternate method of administration, such as direct injection into the joint, may still prove to be effective as a treatment for OA. Indeed, Syk inhibitors are currently in development by a number of pharmaceutical companies.

## Abbreviations

BCP: Basic calcium phosphate
DAMP: Damage-associated molecular pattern
DC: Dendritic cells
ELISA: Enzyme-linked immunosorbent assay
HA: Hydroxyapatite
LPS: Lipopolysaccharide
MAPK: Mitogen-activated protein kinase
MATS: Membrane affinity-triggered signalling
M-βCD: Methyl-β-cyclodextrin
MMP: Matrix metalloprotease
MSU: Monosodium urate
OA: Osteoarthritis
PCR: Polymerase chain reaction
PI3K: Phosphoinositide-3 kinase
Syk: Spleen tyrosine kinase
TIMP: Tissue inhibitor of metalloprotease
